# A 5-mm open umbilical entry technique for laparoscopic gynecologic surgery

**DOI:** 10.1007/s00423-026-04172-6

**Published:** 2026-08-08

**Authors:** Carolyn Robb, Lauren Tetelbaun, Patricia Xu, Chloe Schreiber, Alexis Greene, Martin Keltz

**Affiliations:** 1 New York Medical College School of Medicine, Valhalla, NY USA; 2 WestMed Reproductive Services, Purchase, NY USA

**Keywords:** laparoscopic entry, open entry technique, peritoneal access, umbilical entry, gynecological laparoscopy

## Abstract

**Background and objectives:**

Laparoscopic entry carries a complication rate of up to 1.3%, including bowel and vascular injury. The Hasson, Veress, and 5-mm open entry techniques differ in approach: Hasson uses a 1–2 cm open incision, Veress involves blind needle access, and the 5-mm open entry technique uses a small umbilical incision with blunt peritoneal entry followed by insertion of a 5-mm bladeless optical trocar. Previously shown to be safe and easily mastered by both attendings and residents, this study re-evaluates the 5-mm technique in a multi-surgeon setting.

**Methods:**

The senior author’s extensive clinical experience with this technique informed its continued use and motivated the present retrospective evaluation of all consecutive cases performed between 2021 and 2025. The technique involves tenting the umbilicus with three tonsil clamps, injecting 1 mL of 0.25% bupivacaine, making a 2–4 mm vertical incision within the umbilical scar that is then stretched to approximately 5 mm with a tonsil clamp, and bluntly dissecting the fascia to expose the mid-umbilical fascia and enter the peritoneal cavity. A 5-mm bladeless optical trocar is advanced under direct visualization, and carbon dioxide insufflation begins once placement is confirmed.

**Results:**

All cases achieved successful entry without conversion to Veress. One patient had a Hasson entry for concurrent cholecystectomy. Omental adhesions were present in 15 cases (7.7%). No entry-related complications occurred.

**Conclusion:**

In this cohort, the 5-mm open entry technique was successfully performed without entry-related complications and demonstrated favorable cosmetic outcomes.

**Supplementary Information:**

The online version contains supplementary material available at https://doi.org/10.1007/s00423-026-04172-6.

## Introduction

Safe and effective abdominal entry is a critical first step in laparoscopic surgery, as complications during this phase can significantly impact patient outcomes. Although rare, laparoscopic entry is associated with a complication rate as high as 1.3%, with bowel and vascular injuries being the most serious [1]. Notably, nearly half of all complications related to laparoscopy occur during peritoneal access and the creation of pneumoperitoneum [2, 3]. The traditional Veress needle approach remains widely used in gynecological surgery due to its speed and familiarity, though it involves blind insertion, which can increase the risk of injury, especially in patients with prior abdominal surgery [4]. Open techniques, such as the Hasson method, offer the advantage of direct visualization but require larger incisions, which can be used for specimen removal, and longer operative times [5]. In 2007, a novel 5-mm open entry technique, implemented to improve safety and cosmesis while avoiding blind instrument placement, was reported [6]. Here, we present an updated report of the 5-mm open entry technique utilized by a gynecological surgeon and partner where specimen removal utilized a cosmetically preferable low two-centimeter suprapubic port.

## Methods

### Study design

We conducted a retrospective review of 195 laparoscopic gynecological surgeries performed by two surgeons from 2021 to 2025. Descriptive statistics were performed using RStudio. Approval for the study was obtained by the institutional review board prior to the initiation of the study.

### Surgical technique

**Fig. 1:**
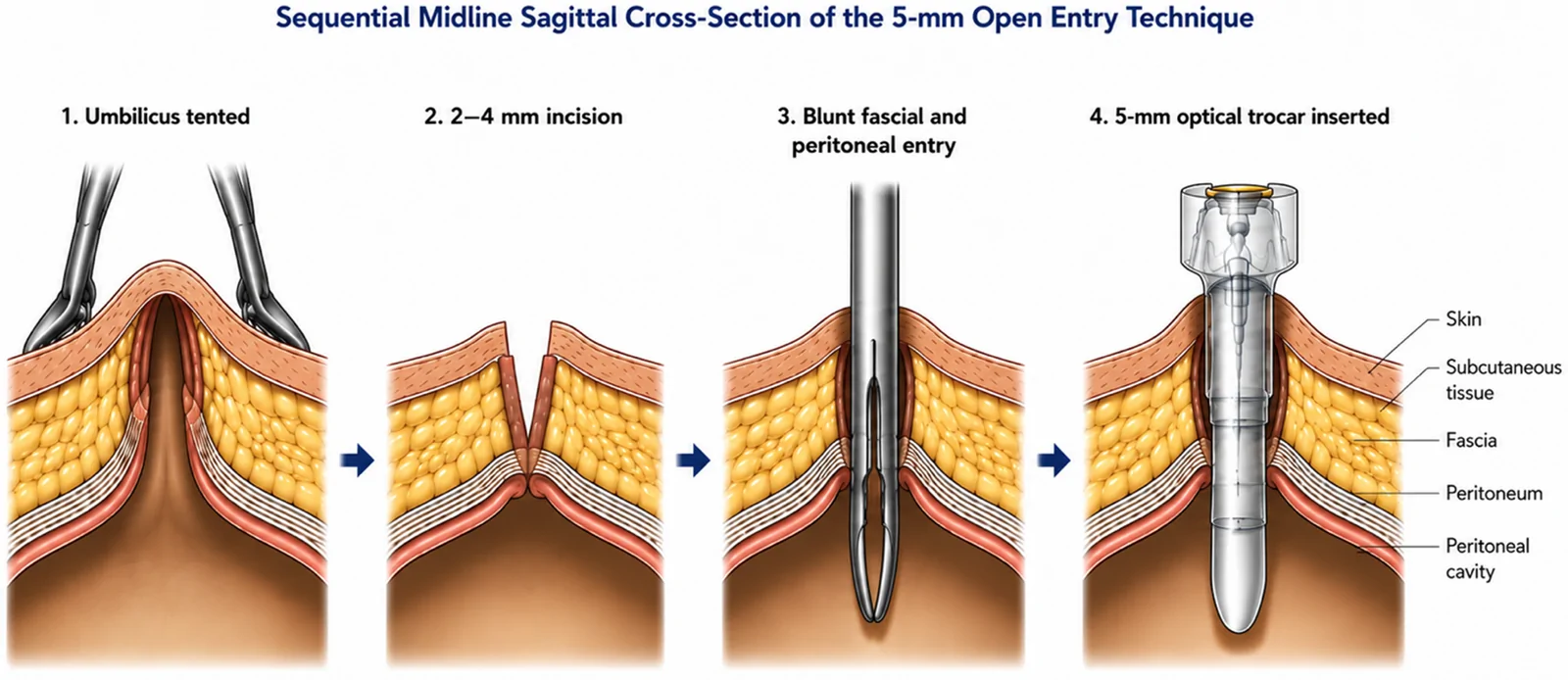
Sequential sagittal cross-sectional schematic of the 5-mm open umbilical entry technique. The illustration demonstrates the key steps of the technique in the midline sagittal plane. From left to right: (1) the umbilicus is elevated using three tonsil clamps to create fascial tenting; (2) a 2–4 mm vertical skin incision is made within the umbilical scar; (3) blunt dissection with a closed tonsil clamp separates the fascial and peritoneal layers, establishing peritoneal access before trocar placement; and (4) a 5-mm bladeless optical trocar is advanced through the already established tract into the peritoneal cavity. The labeled cross-section illustrates the relationships between the skin, subcutaneous tissue, fascia, peritoneum, and peritoneal cavity

**Fig. 2:**
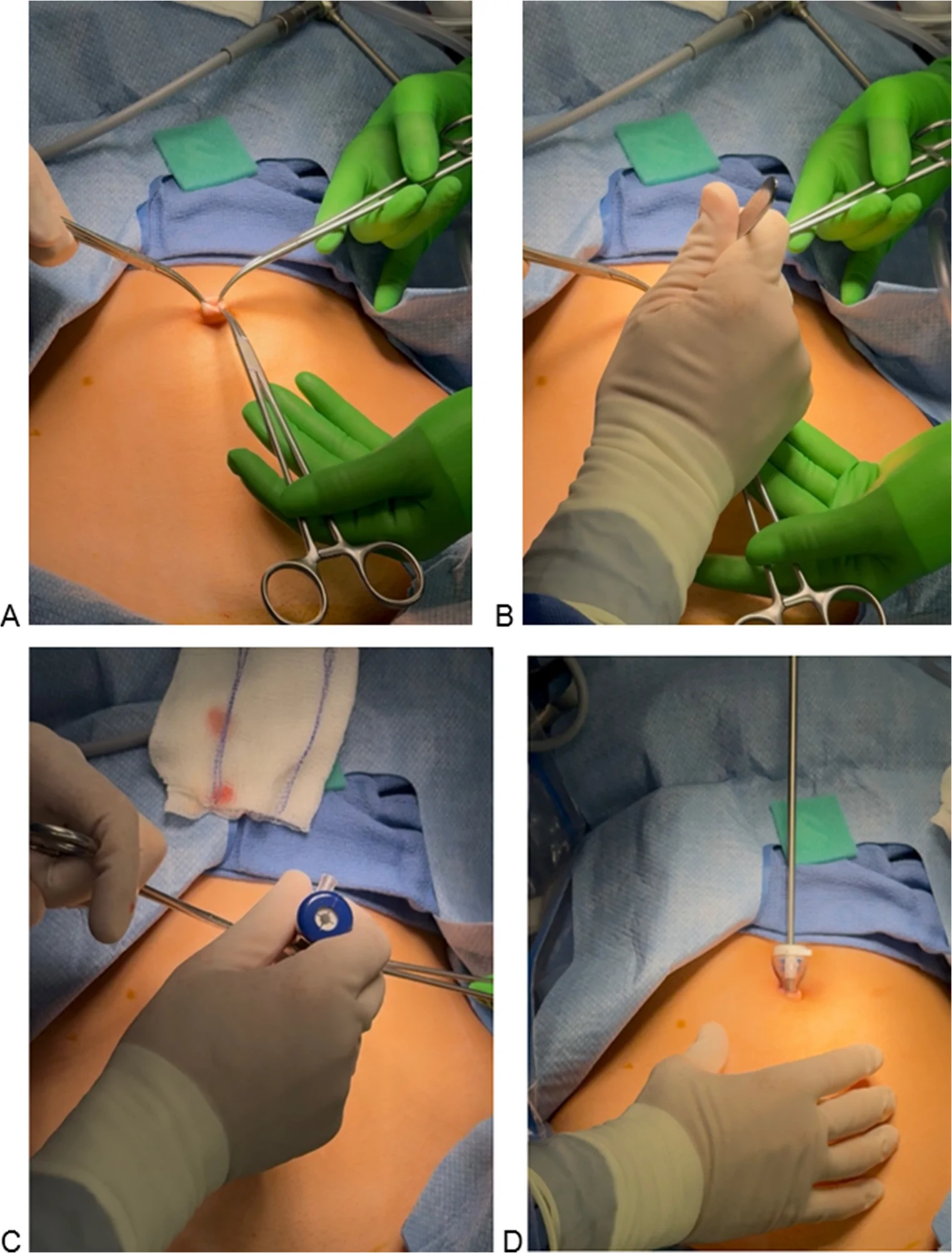
(**A**) The umbilicus is grasped and elevated using three tonsil clamps (**B**) A No. 11 blade scalpel is used to make a 2–4 mm vertical skin incision within the umbilicus, between the elevated clamps (**C**) Insertion of bladeless optical trocar (**D**) 5-mm laparoscope placed through the bladeless optical trocar to visualize the intraabdominal cavity

The patient is placed in a dorsal lithotomy position and prepped and draped in usual sterile fashion. A sagittal cross-sectional schematic of the technique is shown in Fig. 1 and illustrates the sequential steps of umbilical tenting, fascial and peritoneal entry, and optical trocar placement (Fig. 1). The umbilicus, just above the inferior margin of the umbilical fold, is grasped and elevated using three tonsil clamps (Fig. 1 and 2A). The assistant administers 1 mL of 0.25% bupivacaine into the umbilicus above the clamp for local anesthesia. A No. 11 blade scalpel is used to make a 2–4 mm vertical skin incision within the umbilicus, between the elevated clamps (Figs. 1 and 2B), which is stretched to fit a 5-mm laparoscope. The third tonsil clamp is then used to bluntly dissect the subcutaneous tissue in a downward spreading motion until the fascial layer is reached. Lateral spreading of the closed tonsil clamp is used to bluntly separate the fascial and peritoneal layers. Entry into the peritoneal cavity is identified by tactile feedback as the closed clamp passes freely through the peritoneum. In patients with increased abdominal wall thickness, blunt dissection is directed more perpendicular to the abdominal wall to facilitate peritoneal entry before insertion of the optical trocar. This is followed by placement of the bladeless optical trocar into the already accessed peritoneal cavity (Fig. 1 C). Trocar placement is confirmed by placing the 5-mm laparoscope through the bladeless optical trocar to visualize the intraabdominal cavity (Figs. 1 and 2D). Pneumoperitoneum is then established using carbon dioxide gas. At the conclusion of the laparoscopic procedure, the 5-mm mid-umbilical incision is closed using an absorbable subcuticular suture.

## Results

The average patient age and BMI were 36.1 ± 5.8 years and 29.0 ± 5.7 kg/m^2^, respectively (Table 1). The rate of prior abdominal surgery, including either prior laparoscopy, laparotomy, or C-section, was 39.2% (Table 2). Surgical indications included endometriosis ablation (75.9%), ovarian cyst or hydrosalpinx (36.4%), tubal infertility (30.3%), and myomectomy for uterine fibroids (16.9%) (Table 3).

**Table 1:** Continuous baseline characteristics of cases (*N* = 195)

Baseline characteristic	Mean (SD) [Range]
Age (years)	36.1 (5.8) [17–54]
BMI (kg/m^2^)	29.0 (5.7) [16.0-47.2]
Gravidity	1.7 (2.2) [0–16]
Parity	0.5 (0.7) [0–3]
Number Prior C-Sections	0.2 (0.5) [0–3]
Number Prior Vaginal Births	0.2 (0.6) [0–3]

**Table 2:** Categorical baseline characteristics of cases (*N* = 195)

Baseline characteristic	Number of cases (%)
Prior laparoscopy	50 (25.8)
Prior laparotomy	9 (4.6)
Prior C-Section	33 (17.0)
Prior abdominal surgery*	76 (39.2)

*Patients who had any prior laparoscopy, laparotomy, or C-Section

The study cohort consisted of 195 consecutive laparoscopic procedures performed during the study period. The 5-mm open entry technique was used in 194 (99.5%) cases. In one patient undergoing a concurrent laparoscopic cholecystectomy, the Hasson technique was used for abdominal entry and specimen removal. Therefore, Tables 1, 2, 3 and 4 describe the overall study cohort, whereas entry-related outcomes of the 5-mm technique pertain to the 194 cases in which it was performed. The 5-mm technique had a 100% success rate of primary peritoneal entry. No cases were converted to Veress needle entry (Table 3). There were no complications related to peritoneal entry. The most reported intraperitoneal adhesions were adnexal (32.3%), paracolic (23.1%), and tubal (18.5%) (Table 3). Only 15 (7.7%) cases had omental adhesions to the anterior abdominal wall.

**Table 3:** Characterization of cases by surgical variables (*N* = 195)

Variable	Number of Cases (%)
5-mm technique	194 (99.5)
Converted to veress needle entry	0 (0.0)
Endometriosis ablation	148 (75.9)
Ovarian cyst or hydrosalpinx	71 (36.4)
Fallopian tube repair	30 (15.4)
Fallopian tube removal	34 (17.4)
Laparoscopic myomectomy	33 (16.9)
Laparoscopic lysis of adhesions	127 (65.1)
Hysteroscopic Biopsy or Polypectomy	148 (75.9)
Hysteroscopic Metroplasty	68 (34.9)
Hysteroscopic Lysis of Adhesions	14 (7.2)
Hysteroscopic Myomectomy	34 (17.4)
Unintended Intraoperative Events	8 (4.1)
Intraperitoneal Adhesions	114 (58.5)
Omental Adhesions to Anterior Abdominal Wall	15 (7.7)
Paracolic Adhesions	45 (23.1)
Adnexal Adhesions	63 (32.3)
Tubal Adhesions	36 (18.5)
Reverted to Open Surgery	0 (0.0)
Hysteroscopic Uterine Perforation	4 (2.0)

**Fig. 3:**
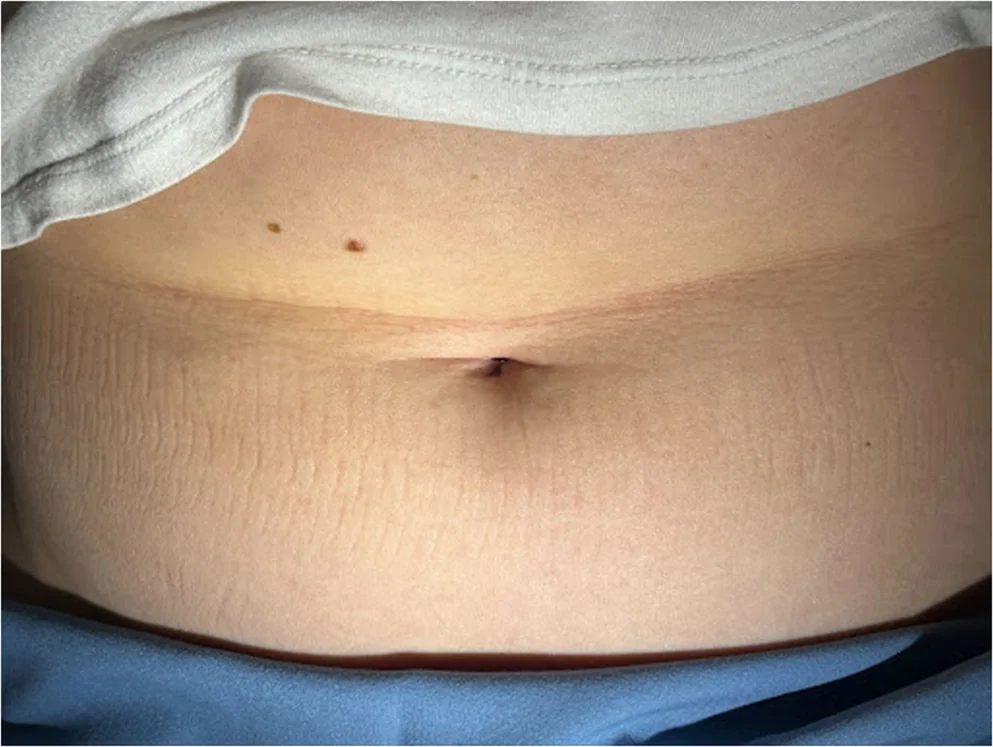
Representative appearance of the umbilicus at 2 weeks following surgery

The rate of unintended intraoperative events was 4.1%, including blunt deserosalization of colon, uterine perforation during hysteroscopy, and bradycardia following insufflation (Table 4). At 2 weeks post-op, the umbilicus demonstrated cosmetic healing with no visible scarring (Fig. 3).

**Table 4:** Unintended intraoperative events

Type of Event	Number of Cases (%)
Blunt deserosalization of bowel	1 (0.5)
Abortion of laparoscopic abdominal cerclage due to poor visualization	1 (0.5)
Errant connection of the methylene blue syringe to the balloon tubing during chromotubation causing application of significant pressure applied to uterus; no significant sequelae	1 (0.5)
Bradycardia post-insufflation	1 (0.5)
Uterine perforation during hysteroscopy	4 (2.0)
Intraoperative events requiring further intervention (i.e. transfusion, readmission, repeat procedures)	0 (0.0)
Total	**8 (4.1)**

## Discussion

Laparoscopic surgery requires safe and reliable access to the peritoneal cavity, and several entry techniques have been developed to minimize the risk of complications. The Veress needle technique, also known as closed entry, is typically performed at the umbilicus or Palmer’s point [7]. It uses a Veress needle and insufflator to create pneumoperitoneum prior to trocar placement. This approach is fast, familiar, and involves a minimal incision. However, because it relies on blind insertion, it carries an increased risk of vascular or bowel injury and is less ideal in patients with a history of abdominal surgery or adhesions [8].

Palmer’s point entry is an alternative site located in the left upper quadrant, 3 cm below the left costal margin in the midclavicular line [8]. This method uses a Veress needle or optical trocar and is particularly useful in patients with midline adhesions, as it avoids umbilical entry [9]. While it is safer in such cases, it carries a risk of injury to the stomach or spleen, especially if the stomach is not decompressed beforehand, and may be technically more difficult in those with enlarged abdominal organs, such as splenomegaly [8, 9].

In contrast, the Hasson technique is an open entry method performed in the periumbilical region, often supraumbilical or infraumbilical, using a Hasson trocar secured with anchoring sutures [5]. After a small incision and direct dissection to the peritoneum, the trocar is placed under direct vision. It is preferred in patients with prior abdominal surgeries due to its reduced injury risk, but it requires a larger incision, is more time-consuming, and necessitates fascial closure and suturing [10].

Broad comparisons of laparoscopic entry techniques underscore ongoing debate about the safest approach. A systematic review found no high-quality evidence favoring the Veress needle, direct trocar, or open techniques universally [11]. Each method has unique advantages and limitations, with surgeon experience and patient-specific anatomy being key determinants of safety [11]. Major complications of laparoscopic entry include vascular or visceral injuries, which are rare but serious. Minor complications include extraperitoneal insufflation, omental injury, subcutaneous emphysema, and failed entry. Although the overall rates of major complications are low for both the Veress needle and Hasson entry techniques, they still occur. One prospective study reported bowel injury rates of 0.1% for Veress needle and 0.08% for Hasson technique [12]. Similarly, a meta-analysis reported vascular injury rates of 0.92% (10/1086) for Veress needle and 0.00% (0/376) for Hasson in non-randomized studies. However, direct randomized comparisons of the two techniques showed no statistically significant difference in major complication rates due to limited evidence [13]. Furthermore, a 2024 single-center retrospective study comparing Hasson and Veress techniques found no significant difference in primary port placement injuries, suggesting both are comparably safe in practice [14]. In contrast, minor complications and failed entry are more frequently associated with the Veress needle than the Hasson or direct trocar techniques [15, 16]. While the Hasson technique is associated with a higher rate of carbon dioxide leakage at the port site, it has significantly lower rates of failed entry and fewer minor injuries [15]. In our experience, clinically significant gas leakage is uncommon with the 5-mm open entry technique because the small incision provides a snug seal around the trocar. On the rare occasion that gas leakage is encountered, a simple skin suture can be placed around the trocar to improve the seal. This maneuver was not required in any patient included in the present study.

In addition to the risk of complications, the cosmetic outcome after surgery is another important factor in selecting a laparoscopic entry technique. A randomized controlled trial reported significantly higher cosmetic survey scores following intraumbilical compared with periumbilical incisions, suggesting that minimizing the umbilical incision may provide cosmetic benefits. In the present series, postoperative photographs demonstrated minimal visible scarring; however, cosmetic outcomes were not formally assessed using validated patient-reported measures [17].

To our knowledge, we are the first to report the use of the 5-mm open entry technique. Advantages of this approach include achieving a small non-visible cosmetic incision, avoiding the Veress needle, and reducing blind access by establishing the tract with blunt dissection before insertion of the optical trocar, which subsequently confirms intraperitoneal placement. However, it does have relatively limited data compared to traditional methods. In the original retrospective study of 65 patients, the 5-mm open entry method demonstrated a 71% success rate without any complications, and failures were easily converted to the standard Veress needle approach. The technique proved to be independent of factors such as prior surgery, BMI, or age, and offered the added benefits of speed and cosmetic outcomes [6]. Although the present study is intentionally limited to the 195 consecutive cases included in this analysis, the technique has been used routinely by the senior author in more than 2,000 laparoscopic procedures over many years. These earlier cases were not included because they originated from a different institution and were documented in archived paper medical records that were not amenable to standardized retrospective data collection. This broader institutional experience was not systematically analyzed and therefore is not presented as study data, but it reflects the maturity of the technique and the extensive clinical experience that informed its standardized application. The present study expands upon the original report published in 2007 by evaluating 195 consecutive cases performed between 2021 and 2025.

Although we are the first to report this 5-mm open entry technique, other modified open entry approaches have been recently described. One such technique utilizing a 5-mm laparoscope involves an 8-mm infraumbilical skin incision, followed by blunt cephalad dissection with a hemostat and trocar placement prior to insufflation [18]. In their 15-year retrospective review of 350 cases, the only major complication was a colon perforation in a patient with unrecognized adhesions. Notably, however, our technique utilizes a smaller 2-4-mm umbilical incision with a direct, single-step open entry, offering enhanced safety, cosmetic outcomes, and procedural simplicity.

Limitations of this study include its single-institution design with two surgeons who have years of familiarity and expertise with the method, limiting the generalizability of our data. Additionally, the study is retrospective in nature, which can introduce bias. In addition, procedural metrics such as entry time, number of insertion attempts, incision length, and gas leakage were not routinely recorded because of the retrospective study design and therefore could not be systematically evaluated. Prospective studies incorporating these standardized outcomes would further characterize the performance of this technique. Finally, as the 5-mm open entry is the only technique employed by these surgeons for gynecological laparoscopy, there is no direct comparison to other entry techniques in terms of complication rates, operative time, or patient outcomes.

While our study has limitations, it has various strengths. First, the study presents data from 195 consecutive laparoscopic cases, reflecting consistent use of the 5-mm open entry technique in routine reproductive gynecologic surgical practice and building upon the cohort of only 65 cases in the original report of this method [6]. Next, the 5-mm technique had a 100% successful peritoneal entry rate when attempted, with no conversions to Veress needle entry. No peritoneal entry complications were reported, even in patients with omental adhesions to the abdominal wall, demonstrating that no entry-related complications occurred in this series when the technique was performed by experienced surgeons. The technique allows for direct visualization during entry, reducing the risks associated with blind insertion, and postoperative follow-up showed cosmetic healing, which is particularly relevant in minimally invasive surgery. Finally, this technique was successful in patients with a wide range of demographic and clinical characteristics, including age, BMI, prior abdominal surgery, presence of abdominal wall adhesions, and surgical indications such as myomectomy for uterine fibroids, ovarian cystectomy, ectopic pregnancy, endometriosis ablation, and tubal infertility (Tables 1, 2 and 3). In patients with increased abdominal wall thickness, the trajectory of blunt dissection was adjusted to a more perpendicular direction toward the peritoneal cavity, rather than angling toward the pelvis. Peritoneal entry was confirmed by tactile feedback with the closed tonsil clamp before advancement of the 5-mm optical trocar through the established tract. No modification of the incision size or instrumentation was required in our cohort, including patients with a BMI of up to 47.2 kg/m².

From a technical standpoint, several considerations may facilitate successful adoption of this approach. Adequate elevation of the umbilical fascia with the tonsil clamp is critical to establish a controlled tract prior to trocar insertion. In patients with obesity or increased abdominal wall thickness, a more perpendicular trajectory toward the peritoneal cavity may improve access. If a satisfactory tract cannot be established or entry is uncertain, surgeons should not hesitate to convert to an alternative entry technique. These technical considerations, together with the accompanying operative images and illustrations, are intended to improve reproducibility and facilitate safe implementation by surgeons interested in adopting the technique.

## Conclusions

In this retrospective series of 195 consecutive laparoscopic gynecologic procedures, the 5-mm open entry technique was successfully performed without entry-related complications and demonstrated favorable cosmetic outcomes. These findings suggest that the technique is a feasible approach for laparoscopic access when performed by experienced surgeons. Prospective, comparative studies are needed to directly compare this technique with established entry methods, including Veress needle and Hasson entry, with respect to complication rates, operative efficiency, and patient outcomes.

## Supplementary material

Below is the link to the electronic supplementary material.

Supplementary Material 1

## Data Availability

All data supporting the findings of this study are available within the paper.

## References

[1] Alkatout I (2017) Complications of Laparoscopy in Connection with Entry Techniques. J Gynecol Surg 33(3):81–91. 10.1089/gyn.2016.0111 PMC5477171 28663686

[2] Watrowski R, Kostov S, Alkatout I (2021) Complications in laparoscopic and robotic-assisted surgery: definitions, classifications, incidence and risk factors – an up-to-date review. Videosurgery Miniinvasive Tech 16(3):501–525. 10.5114/wiitm.2021.108800 PMC8512506 34691301

[3] Härkki-Sirén P, Kurki T (1997) A nationwide analysis of laparoscopic complications. Obstet Gynecol 89(1):108–112. 10.1016/s0029-7844(96)00390-0 8990449

[4] Lécuru F, Leonard F, Jais JP, Rizk E, Robin F, Taurelle R (2001) Laparoscopy in Patients with Prior Surgery: Results of the Blind Approach. JSLS 5(1):13–16 PMC3015404 11303989

[5] Kumar S, Dubey IB, Aggarwal VC, Soni RK Evaluation of Open (Hasson’s) and Closed (Veress) Technique of Intraperitoneal Access for Creation of Pneumoperitoneum in Laparoscopic Surgery. Cureus 16(2):e54770. 10.7759/cureus.54770 PMC10961137 38524074

[6] Keltz MD, Lang J, Berin I (2007) A 5-mm Open-entry Technique Achieves Safe, Single-step, Cosmetic Laparoscopic Entry. JSLS 11(2):195–197 PMC3015733 17761079

[7] Veress Needle - an overview | ScienceDirect Topics. Accessed June 25 (2025) https://www.sciencedirect.com/topics/medicine-and-dentistry/veress-needle

[8] Thepsuwan J, Huang KG, Wilamarta M, Adlan AS, Manvelyan V, Lee CL (2013) Principles of safe abdominal entry in laparoscopic gynecologic surgery. Gynecol Minim Invasive Ther 2(4):105–109. 10.1016/j.gmit.2013.07.003

[9] Granata M, Tsimpanakos I, Moeity F, Magos A (2010) Are we underutilizing Palmer’s point entry in gynecologic laparoscopy? Fertil Steril 94(7):2716–2719. 10.1016/j.fertnstert.2010.03.055 20452584

[10] Krishnakumar S, Tambe P (2009) Entry Complications in Laparoscopic Surgery. J Gynecol Endosc Surg 1(1):4–11. 10.4103/0974-1216.51902 PMC3304260 22442503

[11] Recknagel JD, Goodman LR (2021) Clinical Perspective Concerning Abdominal Entry Techniques. J Minim Invasive Gynecol 28(3):467–474. 10.1016/j.jmig.2020.07.010 32712324

[12] Dunne N, Booth MI, Dehn TCB (2011) Establishing pneumoperitoneum: Verres or Hasson? The debate continues. Ann R Coll Surg Engl 93(1):22–24. 10.1308/003588411X12851639107557 PMC3293265 21054924

[13] Ahmad G, Baker J, Finnerty J, Phillips K, Watson A (2019) Laparoscopic entry techniques. Cochrane Database Syst Rev 2019(1):CD006583. 10.1002/14651858.CD006583.pub5 PMC6353066 30657163

[14] Syed IN, Syed NN, Naseem R, Singh-Ranger D (2024) The Hasson Versus Veress Trocar Wars: Determining the Safety Index of Laparoscopic Surgical Entry Techniques. Cureus 16(11):e74073. 10.7759/cureus.74073 PMC11661874 39712754

[15] Cornette B, Berrevoet F (2016) Trocar Injuries in Laparoscopy: Techniques, Tools, and Means for Prevention. A Systematic Review of the Literature. World J Surg 40(10):2331–2341. 10.1007/s00268-016-3527-9 27146054

[16] Jiang X, Anderson C, Schnatz PF (2012) The safety of direct trocar versus Veress needle for laparoscopic entry: a meta-analysis of randomized clinical trials. J Laparoendosc Adv Surg Tech A 22(4):362–370. 10.1089/lap.2011.0432 22423957

[17] Lee JS, Hong TH (2016) Intraumbilical versus periumbilical incision in laparoscopic cholecystectomy: A randomized controlled trial. Int J Surg Lond Engl 33:83–87. 10.1016/j.ijsu.2016.07.071. Pt 27494996

[18] Pryor KP, Hurd WW (2016) Modified Open Laparoscopy Using a 5-mm Laparoscope. Obstet Gynecol 127(3):535–538. 10.1097/AOG.0000000000001202 26855100

