# Supplementary material for: A 5-mm open umbilical entry technique for laparoscopic gynecologic surgery
Source: Langenbecks Arch Surg. 2026 Aug 8;411(1):304. doi: 10.1007/s00423-026-04172-6 (PMC13736458; doi:10.1007/s00423-026-04172-6)
Supplement: Supplementary file 1 [file 423_2026_4172_MOESM1_ESM.pdf]

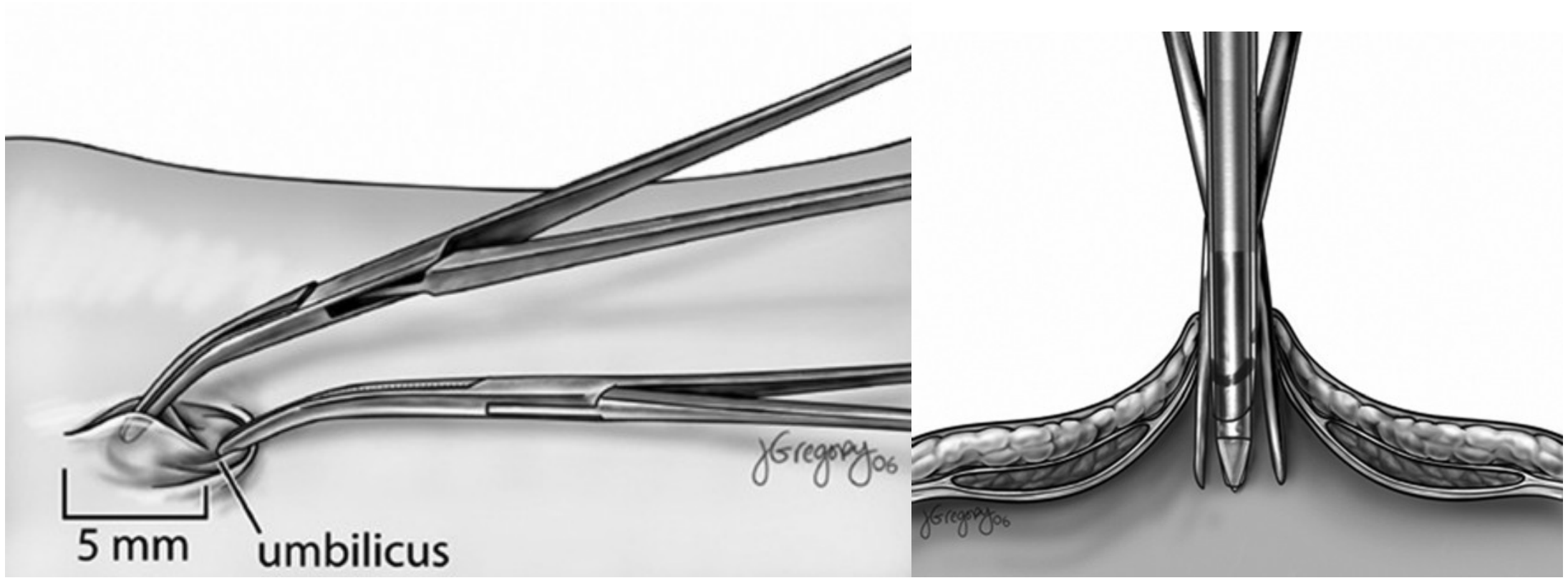

Supplementary Figure S1. Original schematic illustrations demonstrating the 5-mm open umbilical entry technique. Reproduced from Keltz MD et al., *JSLS*, 2007, under the Creative Commons Attribution–NonCommercial–NoDerivatives 3.0 License. No modifications have been made.
